# Sarimini from Vietnam: first record of the genus *Tetrichina* with a new species, and a new species of *Dactylissus* (Hemiptera, Fulgoromorpha, Issidae)

**DOI:** 10.3897/zookeys.1273.170785

**Published:** 2026-03-17

**Authors:** Jérôme Constant, Thai-Hong Pham, Tuong Cat Thi Truong, Hoai Thu Thi Nguyen

**Affiliations:** 1 Royal Belgian Institute of Natural Sciences, O.D. Taxonomy & Phylogeny - Entomology, Vautier street 29, B-1000 Brussels, Belgium Hue and Vietnam and Graduate School of Science and Technology, Vietnam Academy of Science and Technology Hanoi Vietnam; 2 Mientrung Institute for Scientific Research, Vietnam National Museum of Nature, VAST, 321 Huynh Thuc Khang, Hue and Vietnam and Graduate School of Science and Technology, Vietnam Academy of Science and Technology, 18 Hoang Quoc Viet, Hanoi, Vietnam Royal Belgian Institute of Natural Sciences, O.D. Taxonomy & Phylogeny - Entomology Brussels Belgium

**Keywords:** Biodiversity, Fulgoroidea, Indochina

## Abstract

Two new planthopper species of the family Issidae, tribe Sarimini are described from Central Vietnam. *Dactylissus sinuatus* Constant & Pham, **sp. nov**. from Hon Ba Nature Reserve in Khanh Hoa Province, represents the second species of the genus *Dactylissus* Gnezdilov & Bourgoin, 2014 so far known only from Hon Ba, and *Tetrichina honbana* Constant & Pham, **sp. nov**. from Hon Ba Nature Reserve, Nui Chua National Park in Khanh Hoa Province and Ta Kou Nature Reserve in Binh Thuan Province, represents the first record of the genus *Tetrichina* Chang & Chen, 2020 in Vietnam. Illustrations of habitus and terminalia of the new species are given as well as a distribution map and photographs of live specimens and their habitat.

## Introduction

With more than 1,100 species distributed in approximately 230 genera (Bourgoin 2025), the family Issidae Spinola, 1839 is a large and globally distributed group of planthoppers (Hemiptera: Fulgoromorpha), representing ~ 8% of all known Fulgoromorpha species. Important progress was achieved in the documentation of new taxa in the recent years but some major regions such as tropical Africa, New Guinea, and Australia remain very poorly documented (Gnezdilov and Fletcher 2010; Gnezdilov 2013; Gnezdilov et al. 2022; Constant and Semeraro 2023). The tribe Sarimini currently counts ~ 160 species in 40 genera, and several species and genera were described / recorded from north and Central Vietnam in the recent years (see Constant and Pham 2024, 2025a, 2025b).

Our study of the material of Issidae in the collections of VNMN and RBINS revealed two new species of Sarimini from central Vietnam, in the genera *Dactylissus* Gnezdilov & Bourgoin, 2014 and *Tetrichina* Chang & Chen, 2020. The genus *Dactylissus* currently contains a single species, *D. armillarius* Gnezdilov & Soulier-Perkins, 2014, described from Hon Ba Nature Reserve in Central Vietnam. The genus *Tetrichina* is only known from Hainan Island in China. It was described to accommodate *T. trihamulata* Chang & Chen, 2020 (Chang et al. 2020), and later the same year a second species was added, *T. fuscovinclum* Wang, Zhang & Bourgoin, 2020 (Wang et al. 2020). Two years later, Gnezdilov (2022) synonymised the genus *Lunatissus* Meng, Qin & Wang, 2020 under *Tetrichina*, and the two species it contained (Zhang et al. 2020): *L. brevis* Che, Zhang & Wang, 2020 as *T. fuscovinclum*, and *L. longus* Che, Zhang & Wang, 2020 as *T. trihamulata*.

The present paper aims to describe two new species of Sarimini from southern central Vietnam and provide some information on their habitat and biology, as a new contribution to the knowledge of the Vietnamese issid fauna. The study area is located at two sites: Hon Ba Nature Reserve and Nui Chua National Park, both situated in Vietnam’s southern central coastal region. At Hon Ba Nature Reserve, the vegetation exhibits a continuous altitudinal zonation, from 150 m to 1,578 m above sea level. The vegetation structure is highly diverse, with distinct landscapes including valley-foot forests, mid-slope forests below 1,000 m, and mountaintop landscapes. The complex mountainous terrain and well-developed hydrological network create an unique system that provides specific microclimatic conditions for various plant species, including both woody and herbaceous plants. Several species reach exceptional sizes compared to other areas. Nui Chua National Park, except for summit areas covered in rainforest, is a prime example of a dry ecosystem, not only in Vietnam but also across Southeast Asia. The flora here is characterised by drought-resistant, thorny plants. Most species are deciduous during the dry season, with low-growing trees that have small, thick leaves, often hairy or serrated. Many species are particularly noted for their abundance of thorns, a key adaptation to reduce transpiration.

## Materials and methods

The specimens were captured by hand using small transparent vials with which they were slowly covered, or by sweeping the lower vegetation, bushes, and lower branches of trees in the forest with nets, along trails.

The photographs of habitats and live specimens were taken with an Olympus Tough 6 camera; some specimens were placed in a fine mesh cage when necessary but in this case, it is mentioned in the caption. The collection specimens were photographed with a Leica EZ4W stereomicroscope with integrated camera, and the images were stacked with CombineZ software and optimized with Adobe Photoshop CS3; all illustrations were made by JC. The distribution maps were produced with SimpleMappr (Shorthouse 2010). The genitalia were extracted after soaking the abdomen in a 10% solution of potassium hydroxide (KOH) at room temperature for ~ 12 h. The pygofer was separated from the abdomen and the aedeagus dissected with a needle blade for examination. The whole was thoroughly rinsed in 70% ethanol, then placed in glycerine for preservation in a tube attached to the pin of the corresponding specimen. The hind wings were glued with white glue onto a small white cardboard rectangle attached to the pin of the corresponding specimen.

The external morphological terminology follows O’Brien and Wilson (1985) and for the terminalia, Bourgoin and Huang (1990), Gnezdilov (2003), and Gnezdilov et al. (2014b). The metatibiotarsal formula gives the number of spines on (side of metatibia) apex of metatibia / apex of first metatarsus / apex of second metatarsus. The terminology of the wing venation follows Bourgoin et al. (2015). The higher classification follows the most recent as published by Gnezdilov et al. (2022).

The measurements are as in Constant (2004) and the following abbreviations are used:

**BB** maximum breadth of the body.

**BF** maximum breadth of the frons.

**BTg** maximum breadth of the tegmen.

**BV** maximum breadth of the vertex.

**BW** maximum breadth of the hind wing.

**LF** length of the frons at median line.

**LT** total length (apex of head to apex of tegmina).

**LTg** length of the tegmen.

**LV** length of the vertex at median line.

**LW** maximum length of the hind wing.

Abbreviations used for the collections:

**RBINS** Royal Belgian Institute of Natural Sciences, Brussels, Belgium.

**VNMN** Vietnam National Museum of Nature, Hanoi, Vietnam.

## Taxonomy

**Family Issidae Spinola, 1839**

**Subfamily Issinae Spinola, 1839**

### Tribe Sarimini

Taxon classificationAnimaliaHemipteraIssidae

Wang, Zhang & Bourgoin, 2016

4CF29BD2-25DE-5083-9036-0BB78AC2A7A7

#### Type genus.

*Sarima* Melichar, 1903.

### Genus Dactylissus

Taxon classificationAnimaliaHemipteraIssidae

Gnezdilov & Bourgoin, 2014

B39FAB19-224A-58BF-86FE-1EBBD16855E1

Dactylissus Gnezdilov & Bourgoin, 2014 in Gnezdilov et al. 2014a: 86. Type species: Dactylissus armillarius Gnezdilov & Soulier-Perkins, 2014, by original designation.

#### Species included

**(with distribution)**.

*Dactylissus armillarius* Gnezdilov & Soulier-Perkins, 2014 (Vietnam, Khanh Hoa Province, Hon Ba Nature Reserve – Gnezdilov et al. 2014a).

*Dactylissus sinuatus* Constant & Pham, sp. nov. (Vietnam, Khanh Hoa Province, Hon Ba Nature Reserve – this study).

### Dactylissus sinuatus

Taxon classificationAnimaliaHemipteraIssidae

Constant & Pham sp. nov.

E4E13DEF-86F7-51F2-991B-36553DA07EDE

https://zoobank.org/E267FDE7-25DC-41D3-9D59-075D893BDE2B

**Figure 1.**
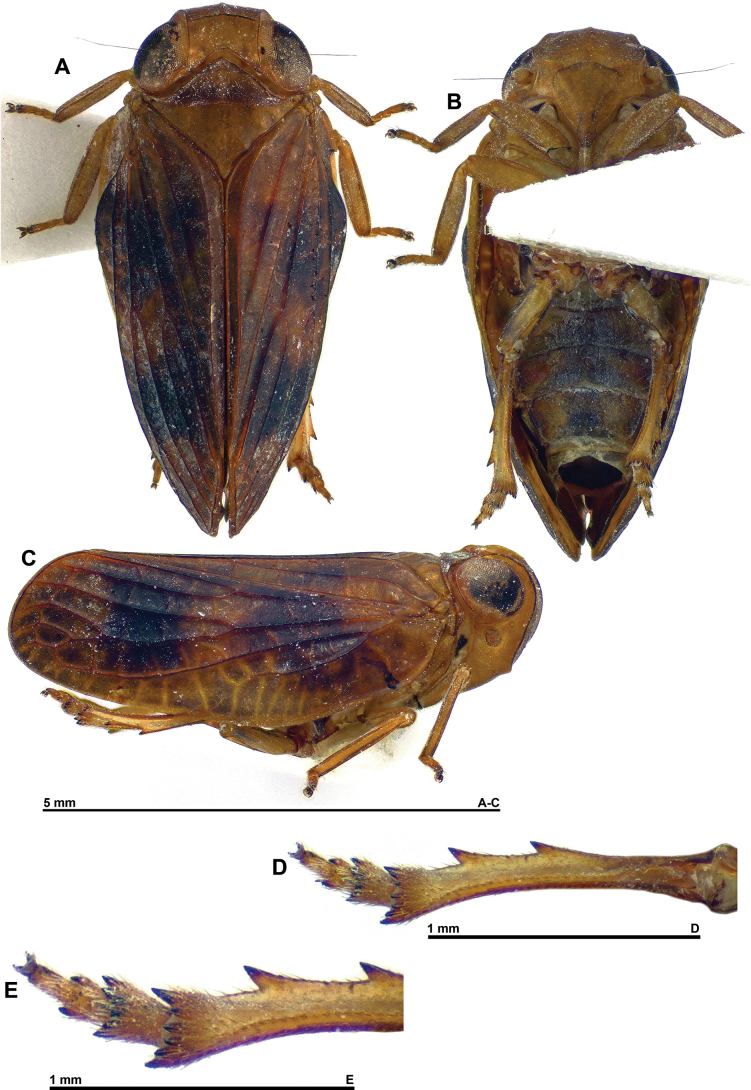
*Dactylissus sinuatus* Constant & Pham, sp. nov., holotype ♂ (VNMN). **A**. Habitus, dorsal; **B**. Habitus, ventral; **C**. Habitus, lateral; **D**. Metatibia and metatarsus, ventral; **E**. Distal portion of metatibia and metatarsus, ventral.

**Figure 2.**
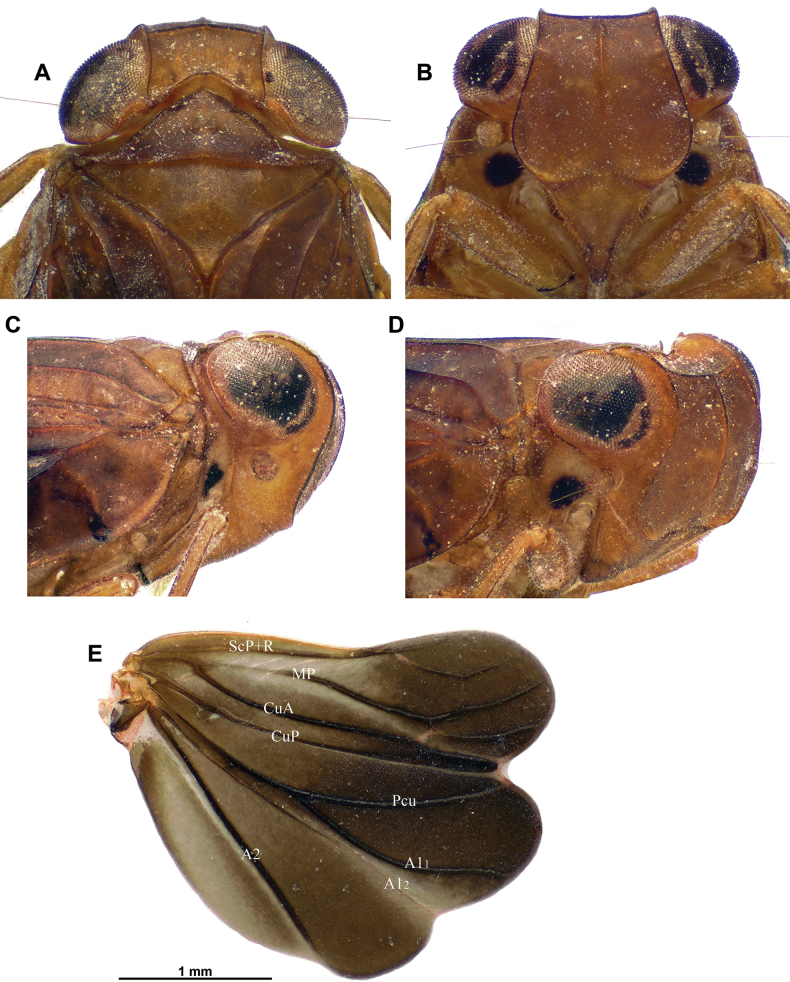
*Dactylissus sinuatus* Constant & Pham, sp. nov., holotype ♂ (VNMN). **A**. Head and thorax, dorsal; **B**. Frons, perpendicular; **C**. Head and thorax, lateral; **D**. Head and thorax, anterolateral; **E**. Right hind wing.

**Figure 3.**
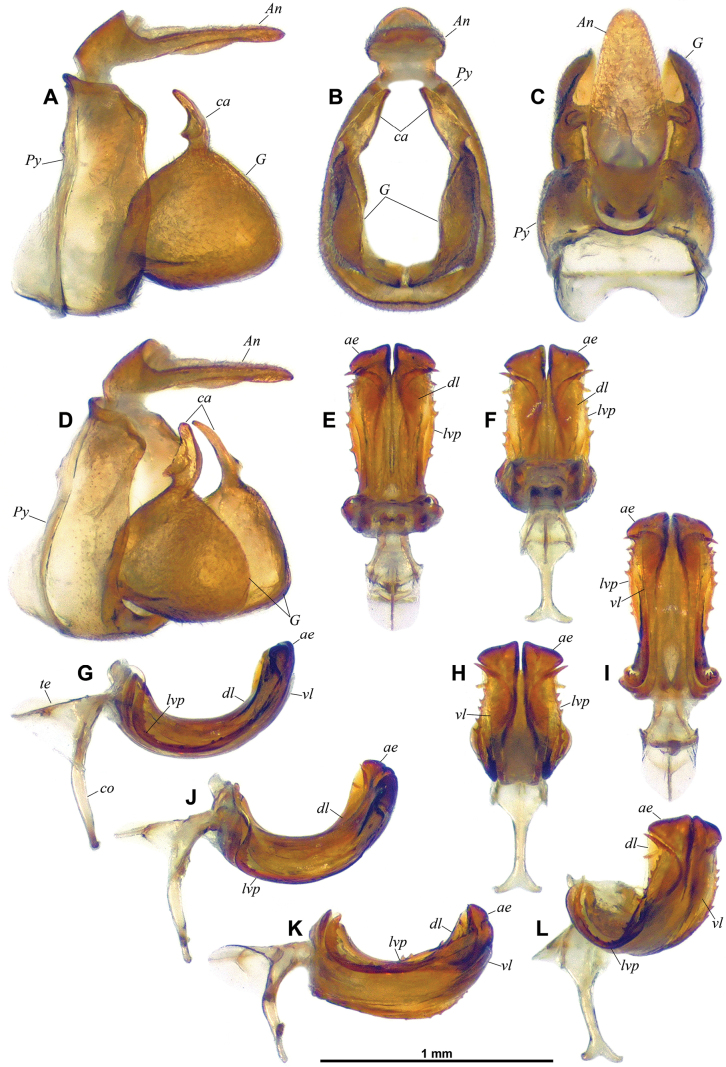
*Dactylissus sinuatus* Constant & Pham, sp. nov., holotype ♂ (VNMN), terminalia. **A–D**. Pygofer, anal tube and gonostyli; **A**. Left lateral; **B**. Caudal; **C**. Dorsal; **D**. Posterolateral; **E–L**. Aedeagus; **E**. Dorsal; **F**. Anterodorsal; **G**. Left lateral; **H**. Posteroventral; **I**. Ventral; **J**. Left laterodorsal; **K**. Left lateroventral; **L**. Posterolateral.

**Figure 4.**
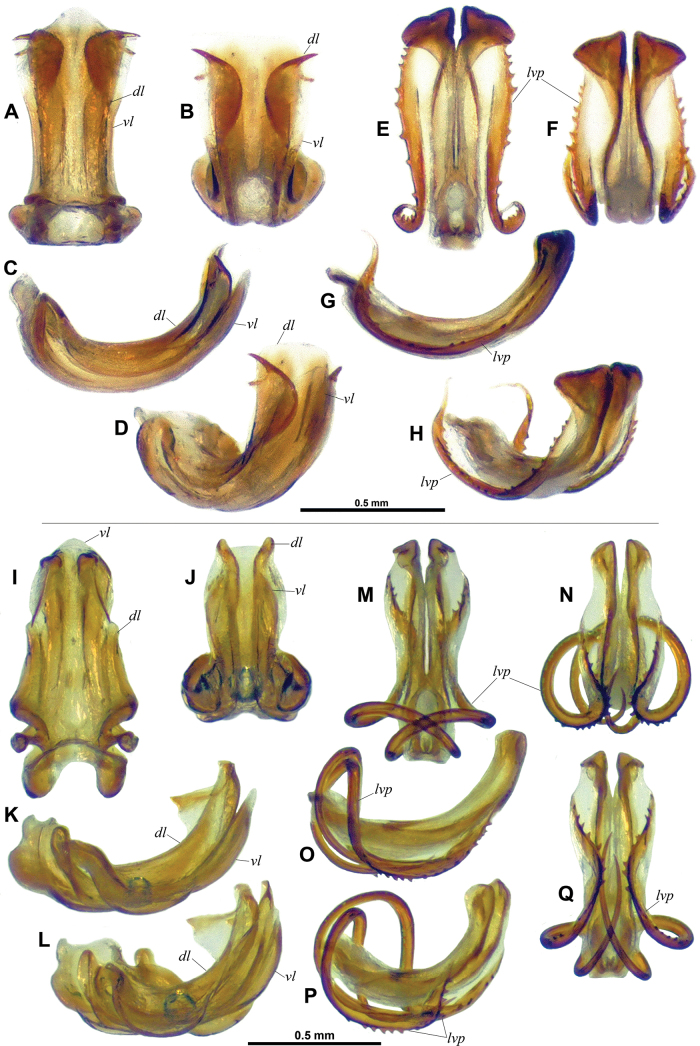
**A–H**. *Dactylissus sinuatus* Constant & Pham, sp. nov., holotype ♂ (VNMN). **A–D**. Periandrium; **A**. Dorsal; **B**. Posteroventral; **C**. Left lateral; **D**. Posterolateral; **E–H**. Aedeagus sensu stricto; **E**. Dorsal; **F**. Posteroventral; **G**. Left lateral; **H**. Posterolateral. **I–Q**. *D. armillarius* ♂ (RBINS). **I–L**. Periandrium; **I**. Dorsal; **J**. Posteroventral; **K**. Left lateral; **L**. Posterolateral; **M–Q**. Aedeagus sensu stricto; **M**. Dorsal; **N**. Posteroventral; **O**. Left lateral; **P**. Posterolateral; **Q**. Ventral.

**Figure 5.**
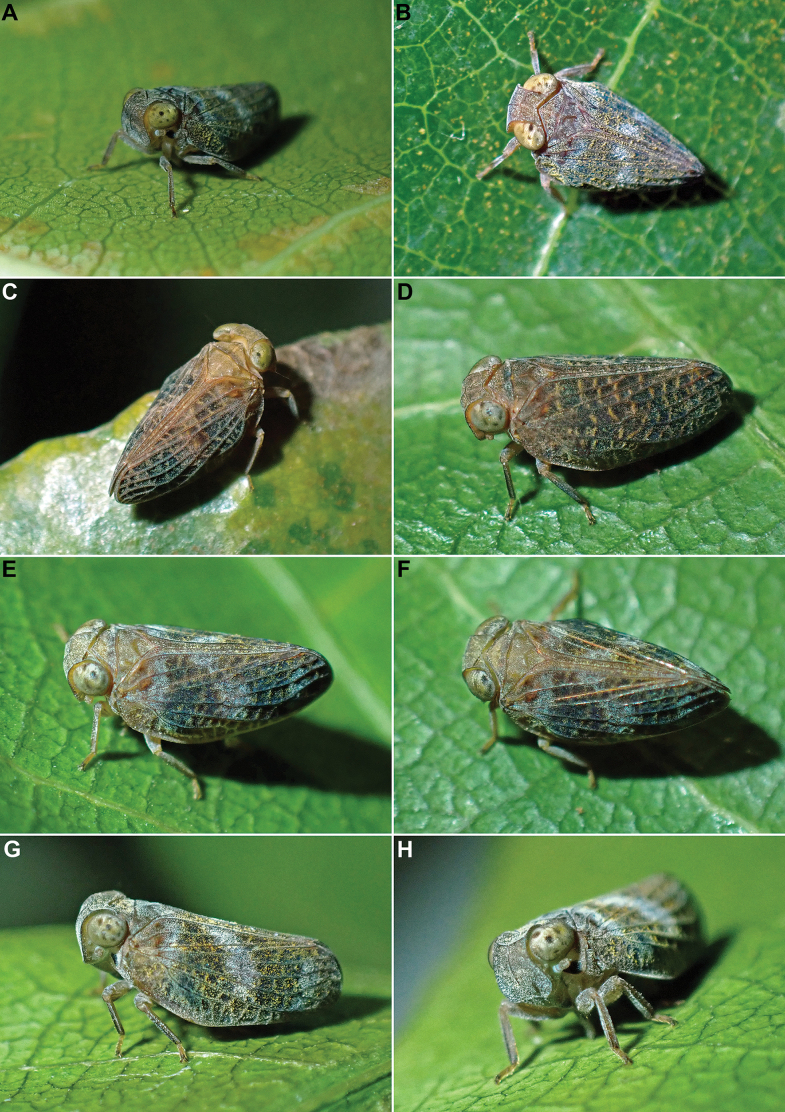
*Dactylissus sinuatus* Constant & Pham, sp. nov. **A–H**. Live specimens in Hon Ba Nature Reserve, 9–11 Oct. 2024.

**Figure 6.**
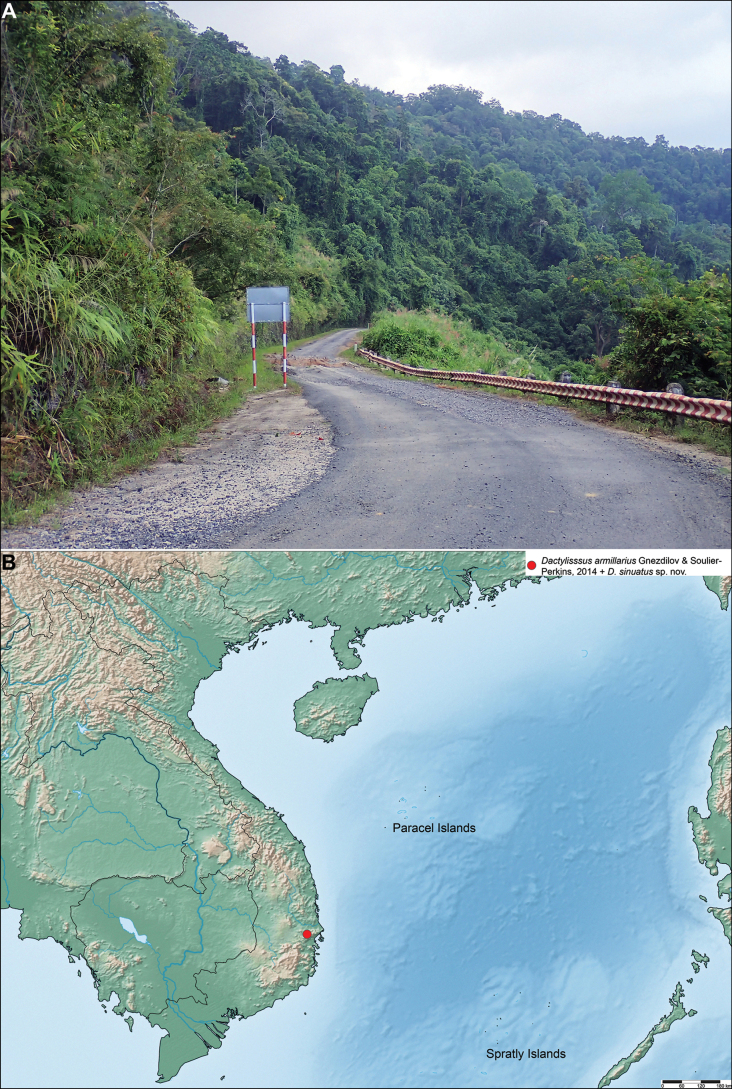
**A**. Habitat of *Dactylissus sinuatus* Constant & Pham, sp. nov. in Hon Ba Nature Reserve, 11 Oct. 2024; **B**. Distribution map of the species of *Dactylissus* Gnezdilov & Bourgoin, 2014.

Figs 1, 2, 3, 4, 5, 6

#### Type material.

***Holotype*** ♂, **Vietnam** • Khanh Hoa Province, Hon Ba Nature Reserve; 12°06'46"N, 108°59'52"E; 300–800 m a.s.l.; 8–11 Oct. 2024; J. Constant, J. Bresseel, L. Semeraro and Hoai T.T. Nguyen leg.; VNMN. ***Paratypes*, Vietnam** • 1 ♂, [paratype of *Dactylissus armillarius* Gnezdilov & Soulier-Perkins, 2014]; Vietnam, Khanh Hoa Province, Hòn Bà massif; 12°6.961'N, 108°58.734'E; 13 Nov. 2013; 850 m; sweeping; Th. Bourgoin leg.; “Mission Hòn Bà MNHN 2013”; EH30881; MNHN • 3 ♂♂, 1 ♀; same data as for holotype; I.G.: 34893; RBINS • 3 ♂♂, 1 ♀; same data as for holotype; VNMN • 3 ♂♂, 2 ♀♀; Khanh Hoa Province, Hon Ba Nature Reserve; 12°07'19"N, 108°56'53"E; 900 m; 11–15 Jul. 2025; J. Constant, J. Bresseel L. Semeraro, Trung Thanh Vu leg.; VNMN • 4 ♂♂, 3 ♀♀; same data as for preceding; I.G.: 35028; RBINS.

#### Diagnosis.

**Figure 7.**
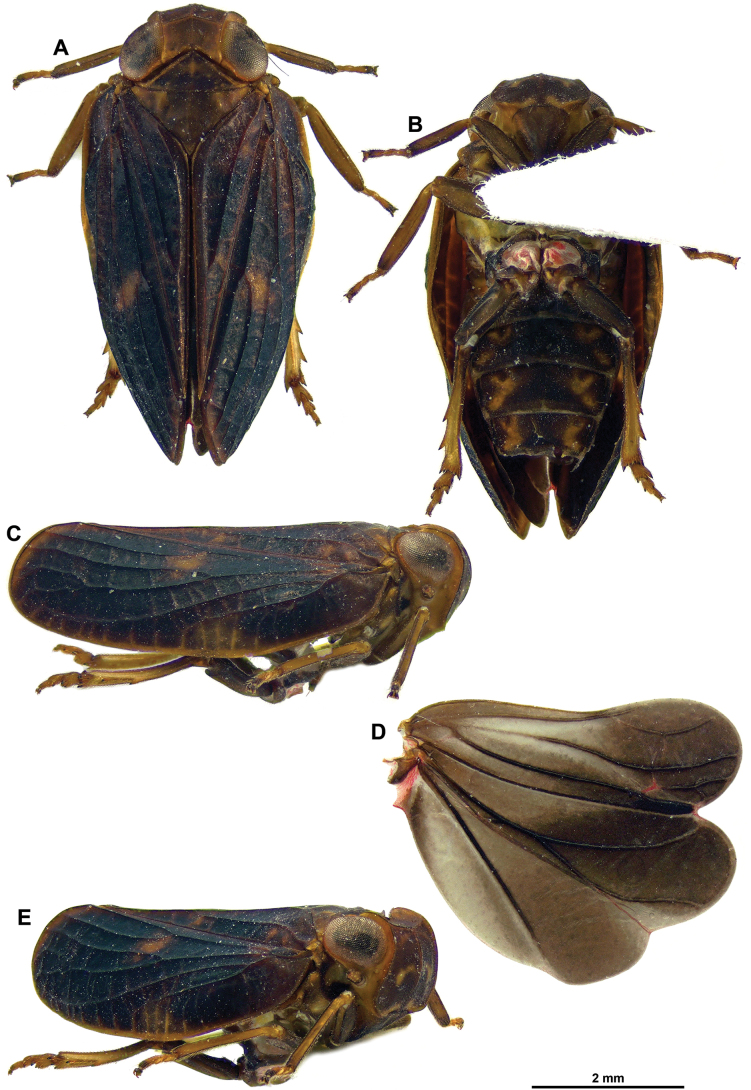
*Dactylissus armillarius* Gnezdilov & Soulier-Perkins, 2014, ♂ (RBINS). **A**. Habitus, dorsal; **B**. Habitus, ventral; **C**. Habitus, lateral; **D**. Hind wing; **E**. Habitus, anterolateral view.

**Figure 8.**
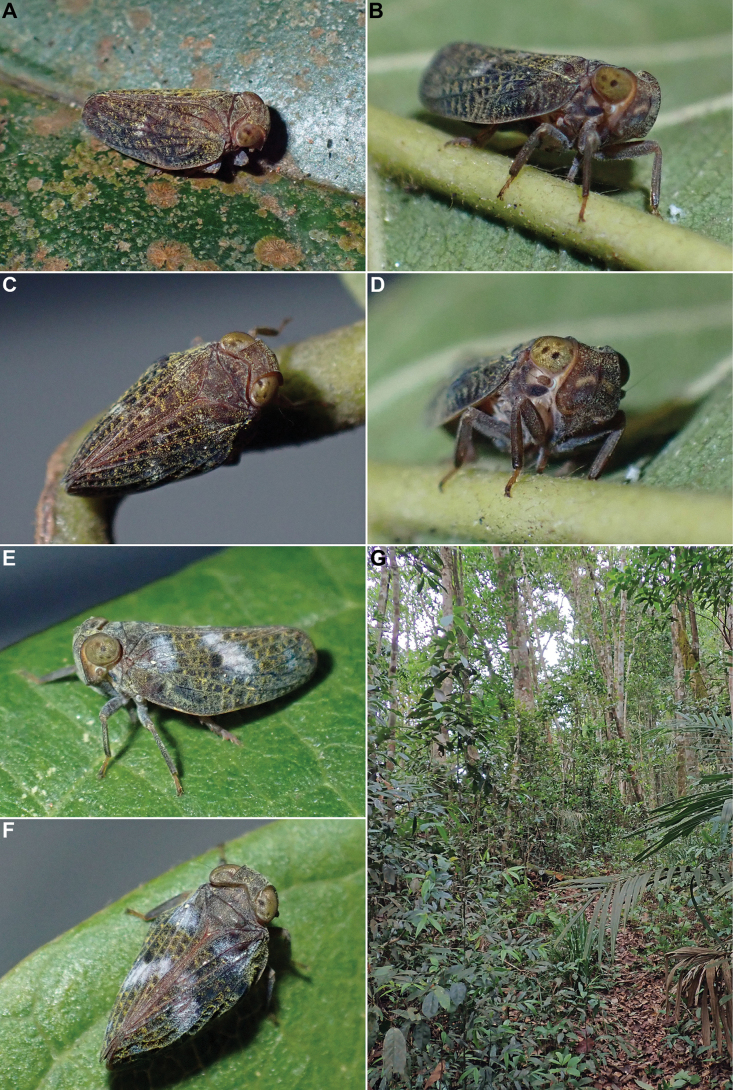
*Dactylissus armillarius* Gnezdilov & Soulier-Perkins, 2014. **A–F**. Live specimens in Hon Ba Nature Reserve, 11 Jul. 2025; **G**. Habitat in Hon Ba Nature Reserve, 1400 m, 11 Jul. 2025.

*Dactylissus sinuatus* Constant & Pham, sp. nov. (Figs 1, 2, 3, 4, 5) is externally similar to *D. armillarius* Gnezdilov & Soulier-Perkins, 2014 (Figs 4I–Q, 7, 8; Gnezdilov et al. 2014a: figs 10–21, 26, 28–33) but it is generally paler, and slightly longer (on average, ♂: 5.6 mm, ♀: 6.0; ♂: 5.15 mm, ♀: 5.85 in *D. armillarius*). The two species can be separated by the following characters of the male terminalia: *D. sinuatus* shows a more elongate anal tube in dorsal view (2.5 × as long as wide; 2.3 × in *D. armillarius*), a distinctly more elongate capitulum of the gonostylus, no distinct subapical lateral process on dorsal lobe of periandrium (large triangular subapical lateral process in *D. armillarius*), the aedeagus bifid with each shaft strongly widening distally to obliquely, sinuately truncate apex (apex of shafts not strongly widening in *D. armillarius*), and the lateroventral processes of the aedeagus ending in a whip-shape, sinuated, way on top of the periandrium (distal portion of lateroventral processes strongly elongate and spiralled around base of the periandrium in *D. armillarius*).

#### Description.

***Measurements and ratios***: LT: ♂ (*n* = 7): 5.6 mm (5.5–5.7); ♀ (*n* = 4): 6.0 (5.8–6.2). LT/BB = 2.1; LTg/BTg = 2.3; LW/BW = 1.2; LV/BV = 0.4; LF/BF = 0.9.

***Head*** (Figs 1A–C, 2A–D). Vertex yellow brown, with obsolete median carina; 2.5 × as broad as long in midline, distinctly constricted in middle; disc concave; anterior margin arched; posterior margin rather deeply concave; all margins distinctly carinate. Frons yellow brown, moderately convex, smooth, sometimes with curved yellowish marking on each side of complete median carina; peridiscal carina obsolete; in perpendicular view, dorsal margin concave and lateral margins distinctly sinuate. Genae yellowish brown (slightly paler than frons and vertex) with anteroventral angle slightly projecting anteriad. Clypeus subtriangular, convex, smooth, not keeled or carinate; anteclypeus yellow brown; postclypeus blackish brown apically. Labium yellow brown with last segment longer than broad, shorter than penultimate. Antennae with scape short, ring-shaped, yellowish, and pedicel bulbous, yellowish with basal third darker.

***Thorax*** (Figs 1 A, C, D, 2A–D). Pronotum yellow brown; ~ 0.6 × as long as mesonotum in midline; anterior margin carinate, distinctly, angularly protruding anteriorly between eyes; posterior margin weakly sinuate; no median carina but with impressed point on each side of median line; paradiscal fields very narrow behind eye, laminate; paranotal lobes broad, pale yellowish with small brown area in dorsal portion, with distinct round black spot behind eye, and with posteroventral angle rounded. Mesonotum yellow brown with obsolete median and sublateral (peridiscal) carinae; smooth, distinctly slightly depressed before scutellum. Tegulae yellow brown.

***Tegmina*** (Figs 1A–C, 6). Yellow brown, usually with two wide transverse dark brown bands not reaching costal margin; in live specimens, pale zones often covered in white waxy (powdery) secretion, secretion golden yellow on dark zones; veins concolourous with background, main veins elevated, and cross-veins weakly raised and often paler (especially along costal margin); distinctly convex, and ~ 1.7 × as long as wide in dorsal view when taken together, with distinct lateral hump including vein ScP+RA around basal 1/3; moderate but distinct, yellowish epipleuron; clavus closed, reaching 4/5 of tegmen length. Venation: ScP+R rather short; ScP+RA rather short, curved towards RP but not fused, and not extending beyond midlength of tegmen; RP unforked, long and sinuate; first fork of MP around midlength of tegmen, MP1 with three terminals; first fork of CuA at approximately the same level; Pcu and A1 fused at ~ 2/3 of clavus length, Pcu+A1 reaching apex of clavus; cross-veins more numerous and more strongly marked along costal margin and in distal 1/2 of tegmen.

***Hind wings*** (Fig. 2E). Well developed, with three distinct lobes (Sarimini type) more or less equal in width; mostly brown. Venation: ScP+R and CuA furcate; MP simple, sinuate; second branch of CuA fused distally with CuP; Pcu and A1 fused on basal 1/2, Pcu unforked and A2 simple; one transverse vein between second branch of ScP+R and MP, and between MP and first branch of CuA.

***Legs*** (Fig. 1A–E). Yellow brown, generally paler than tegmina; distal portion of metafemora and basal portion of metatibiae darkened; all spines of posterior legs black apically. Anterior and median legs slightly flattened dorsoventrally, tibiae more slender than corresponding femora; posteroventral margin of pro- and mesofemora with row of minute teeth; pro- and mesotarsi rather elongate. Metatibiae with two lateral spines in distal 1/2, and seven apical spines. Metatarsi rather short with first segment shorter than combined length of remaining segments. First metatarsomere with two latero-apical and seven intermediate spines. Metatibiotarsal formula: (2) 7 / 9 / 2.

***Abdomen*** (Fig. 1B). Yellowish with wide, darker band in middle.

***Terminalia*** ♂ (Figs 3, 4). Pygofer (Fig. 3A–D, *Py*) short, ~ 2.8 × as high as long at midheight in lateral view; in lateral view, posterior margin broadly rounded with small but distinct rounded lobe protruding posteriad in dorsal portion, and dorsal margin oblique and excavate; in caudal view suboval, 1.4 × as high as wide; dorsally abruptly, deeply notched. Gonostyli (Fig. 3A–D, *G*) large, convex, with anterodorsal margin rounded, then abruptly upcurved at base of capitulum; ventral margin straight along most of length; posterior margin broadly rounded in lateral view and angular (obtuse angle) towards base of capitulum; capitulum (Fig. 3A, B, D, *ca*) elongate, digitiform, strongly projecting dorsad and with short but distinct neck, in lateral view curved anterodorsad with roundly pointed apex and with basilateral laminate process projecting cephalad, in caudal view slightly directed mesad and with basilateral laminate process directed lateroventrad, forming a short hook. Anal tube (Fig. 3A–D, *An*) dorsoventrally flattened, and sub-lanceolate, weakly grooved medially beyond anal opening (in basal 1/4), rather elongate, ~ 2.5 × as long as wide in dorsal view, more or less evenly narrowing along distal 2/3 towards rounded apex; in lateral view abruptly narrowing at anal opening, then more or less straight. Aedeagus (Figs 3E–L, 4, *ae*) symmetrical, distinctly curved posterodorsad in lateral view. Ventral lobe of periandrium (Figs 3G–I, 3K, 3L, 4A–D, *vl*) laminate, spatulate with apical margin roundly lanceolate, and shorter and wider (along most length) than dorsal lobe. Dorsal lobe of periandrium (Figs 3E–G, 3J–L, 4A–D, *dl*) moderately expanded into lamina lateroventrally, then slightly widening towards roundly truncate apex; in distal portion, laminate lateral process folded on ventral side between dorsal and ventral lobes, forming an incomplete furrow lodging aedeagus, two strong lateral teeth at laterodistal angle. Aedeagus (Figs 3E–L, 4E–H, *ae*) distinctly surpassing dorsal and ventral lobes of periandrium, bifid, each shaft strongly widening distally to obliquely, sinuately truncate apex; elongate lateroventral processes (Figs 3E–L, 4E–H, *lvp*) arising subapically, curved ventrocephalad (following curvature of aedeagus) and with external margin distinctly dentate, reaching to base of aedeagus, distal portion partly concealed in groove between dorsal and ventral lobes of periandrium, then upcurved to apical portion without teeth, tapering (whip-shaped), and sinuate, with end directed cephalad on top of dorsal lobe of periandrium. Connective (Fig. 3G, *co*) well developed, corpus connective long, weakly curved in middle portion in lateral view, tectiductus (Fig. 3G, *te*) well developed, conical with dorsal crista and wide anterior foramen.

#### Etymology.

The species epithet *sinuatus* (adj., Latin) means sinuate and refers to the shape of the distal portion of the lateroventral process of the aedeagus.

#### Biology.

The specimens were found sitting on leaves (smooth, not hairy) and small branches of lower vegetation and bushes along the road and forest trails, from which they were collected mostly by sweeping, at altitudes from 300 to 900 m a.s.l. (Figs 5, 6A).

#### Distribution.

Vietnam, Khanh Hoa Province, Hon Ba Nature Reserve (Fig. 6B).

### Dactylissus armillarius

Taxon classificationAnimaliaHemipteraIssidae

Gnezdilov & Soulier-Perkins, 2014

1F76025C-1A74-50AA-A805-00D46C02DB0E

Figs 4I–Q, 6B, 7, 8

Dactylissus armillarius Gnezdilov & Soulier-Perkins, 2014 in Gnezdilov et al. 2014a: 88, figs 10–21, 26, 28–33.

#### Material examined.

Vietnam • 4 ♂♂, 5 ♀♀; Khanh Hoa Province, Hon Ba Nature Reserve; 12°07'19"N 108°56'53"E; 1400 m a.s.l.; 11–15 Jul. 2025; J. Constant, J. Bresseel, L. Semeraro, Trung Thanh Vu leg; I.G.: 35028; RBINS • 3 ♂♂, 4 ♀♀; same data as for preceding; VNMN • 1 ♀; Khanh Hoa Province, Hon Ba Nature Reserve; 12°07'19"N, 108°56'53"E; 1300–1400 m a.s.l.; 8–9 Oct. 2024; J. Constant, J. Bresseel, L. Semeraro and Hoai T.T. Nguyen leg.; VNMN • 1 ♀; same data as for preceding; I.G.: 34893; RBINS.

#### Diagnosis.

See under *D. sinuatus* Constant & Pham, sp. nov.

#### Notes.

A male paratype of *D. armillarius* was dissected in the course of the present study and found to belong to *D. sinuatus* Constant & Pham sp. nov.; hence, it was included as a paratype of *D. sinuatus*. This specimen was also illustrated in Gnezdilov et al. (2014a: fig. 27).

#### Biology.

The specimens were found sitting on leaves (smooth, not hairy) and small branches of lower vegetation and bushes along a forest trail, from which they were collected mostly by sweeping, at altitudes from 1300 to 1400 m a.s.l. (Fig. 8).

### Genus Tetrichina

Taxon classificationAnimaliaHemipteraIssidae

Chang & Chen, 2020

44501C19-AEC5-5323-BC06-90AC3191FD49

Tetrichina Chang & Chen, 2020 in Chang et al. 2020: 39. Type species by original designation: Tetrichina trihamulata Chang & Chen, 2020.
Lunatissus Meng, Qin & Wang, 2020 in Zhang et al. 2020: 496. Type species by original designation: Lunatissus brevis Che, Zhang & Wang, 2020 (synonymised by Gnezdilov 2022: 48).

#### Species included

**(with distribution)**.

*Tetrichina fuscovinclum* Wang, Zhang & Bourgoin, 2020 (China (Hainan) – Wang et al. 2020).

= *Lunatissus brevis* Che, Zhang & Wang, 2020 (synonymised by Gnezdilov 2022: 49).

*Tetrichina honbana* Constant & Pham, sp. nov. (southern central Vietnam).

*Tetrichina trihamulata* Chang & Chen, 2020 (China (Hainan) – Chang et al. 2020)

= *Lunatissus longus* Che, Zhang & Wang, 2020 (synonymised by Gnezdilov 2022: 49).

### Tetrichina honbana

Taxon classificationAnimaliaHemipteraIssidae

Constant & Pham sp. nov.

CC01AE87-CB16-57DA-A683-00F777350BC0

https://zoobank.org/7555359B-E3F4-4F43-8423-9A17B0A35C8B

**Figure 9.**
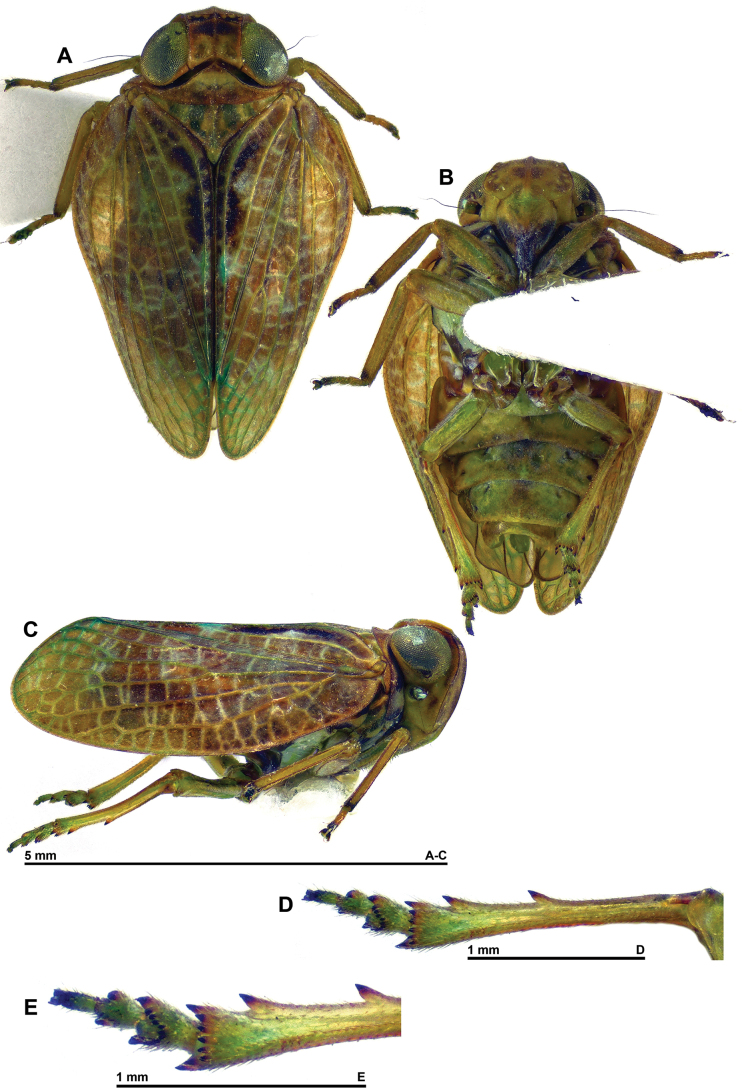
*Tetrichina honbana* Constant & Pham, sp. nov., holotype ♂ (VNMN). **A**. Habitus, dorsal; **B**. Habitus, ventral; **C**. Habitus, lateral; **D**. Metatibia and metatarsus, ventral; **E**. Distal portion of metatibia and metatarsus, ventral.

**Figure 10.**
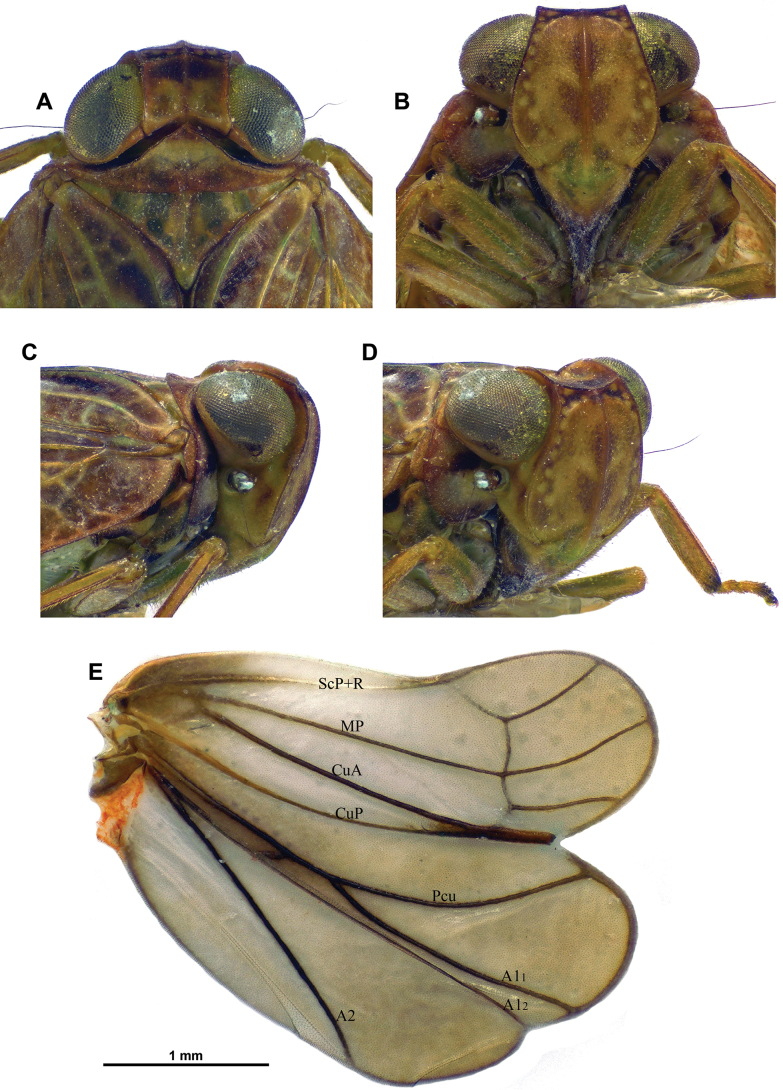
*Tetrichina honbana* Constant & Pham, sp. nov., holotype ♂ (VNMN). **A**. Head and thorax, dorsal; **B**. Frons, perpendicular; **C**. Head and thorax, lateral; **D**. Head and thorax, anterolateral; **E**. Right hind wing.

**Figure 11.**
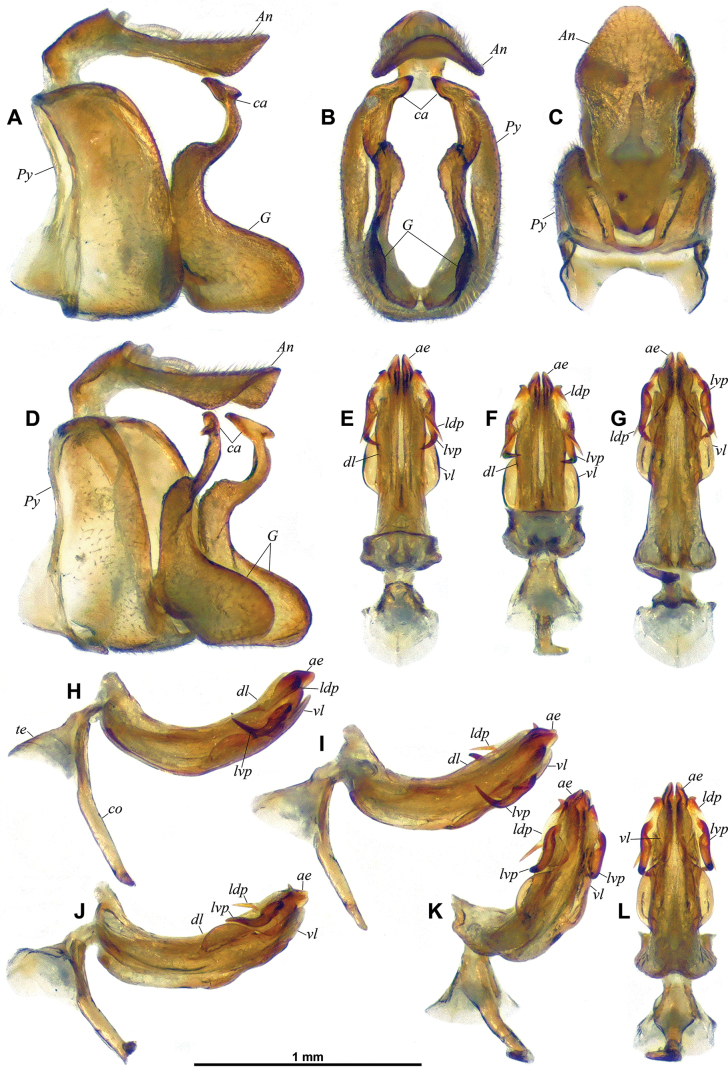
*Tetrichina honbana* Constant & Pham, sp. nov., holotype ♂ (VNMN), terminalia. **A–D**. Pygofer, anal tube and gonostyli; **A**. Left lateral; **B**. Caudal; **C**. Dorsal; **D**. Posterolateral. **E–L**. Aedeagus; **E**. Dorsal; **F**. Anterodorsal; **G**. Ventral; **H**. Left lateral; **I**. Left laterodorsal; **J**. Left lateroventral; **K**. Posterolateral; **L**. Posteroventral.

**Figure 12.**
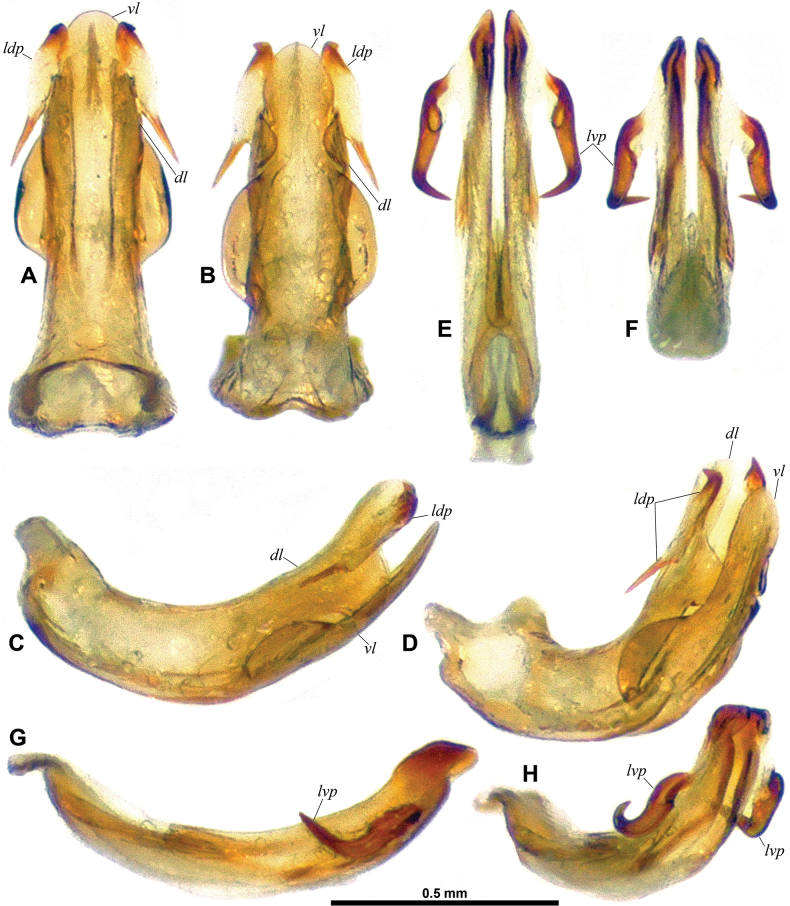
*Tetrichina honbana* Constant & Pham, sp. nov., holotype ♂ (VNMN). **A–D**. Periandrium; **A**. Dorsal; **B**. Posteroventral; **C**. Left lateral; **D**. Posterolateral. **E–G**. Aedeagus sensu stricto; **E**. Dorsal; **F**. Posteroventral; **G**. Left lateral; **H**. Posterolateral.

**Figure 13.**
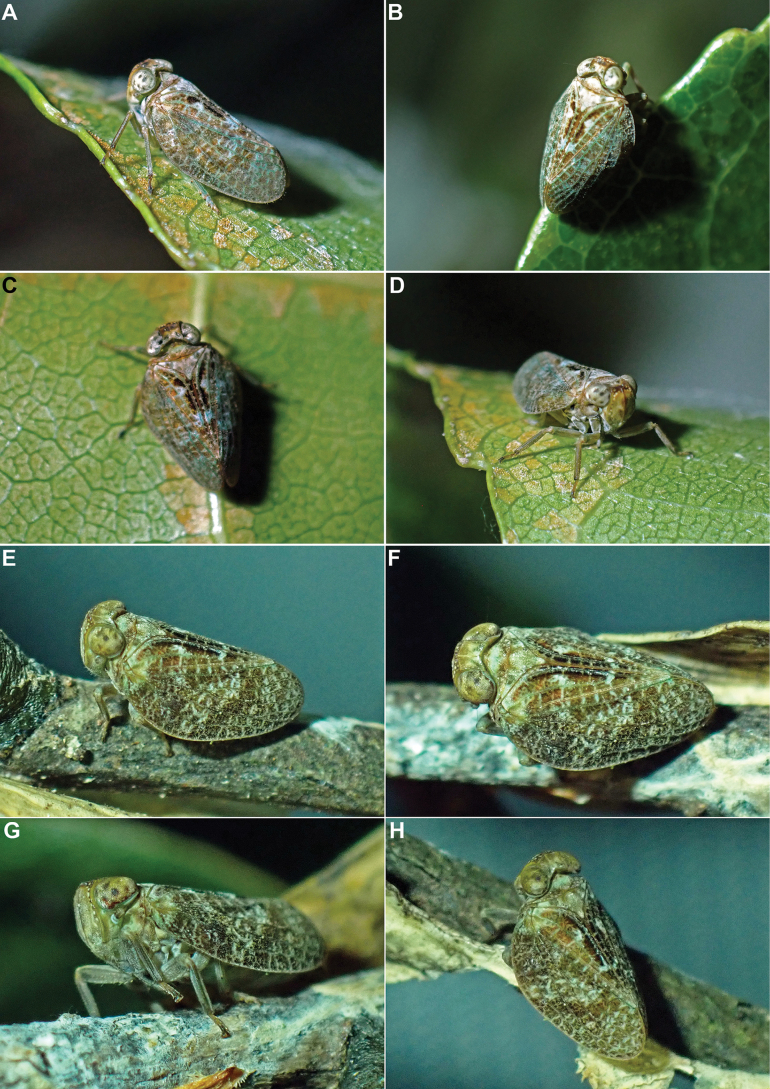
*Tetrichina honbana* Constant & Pham, sp. nov., live specimens. **A–D**. In Hon Ba Nature Reserve, 10 Oct. 2024; **E–H**. In Nui Chua National Park, 4 Oct. 2024.

**Figure 14.**
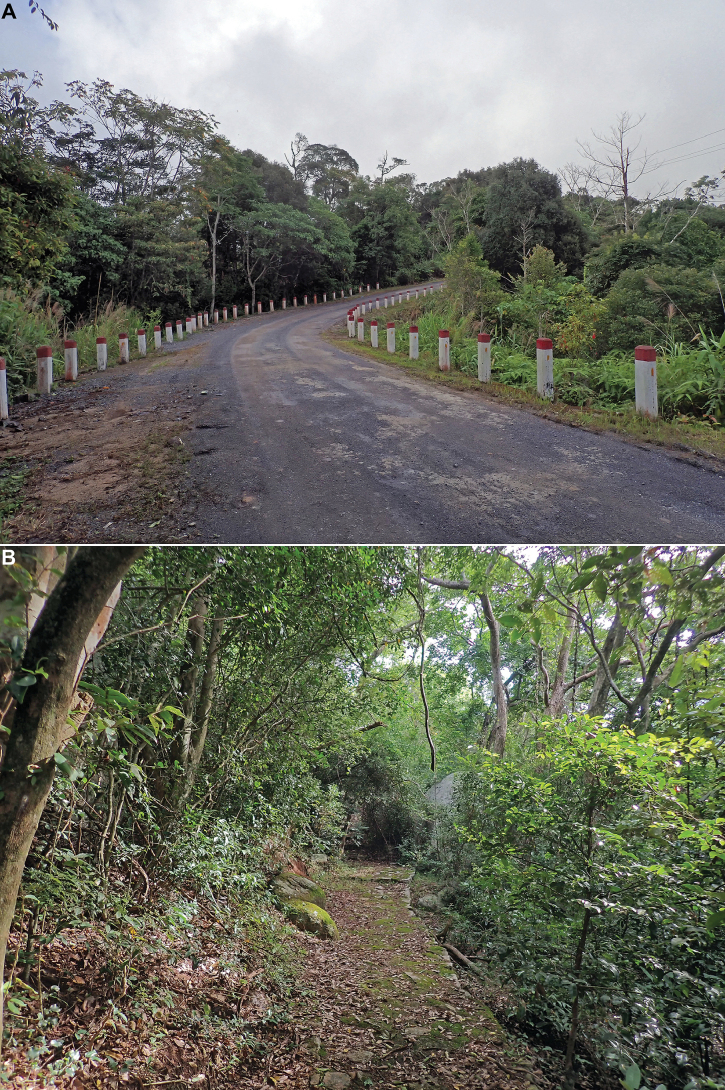
Habitat of *Tetrichina honbana* Constant & Pham, sp. nov. **A**. In Hon Ba Nature Reserve, 9 Oct. 2024; **B**. In Nui Chua National Park, 4 Oct. 2024.

**Figure 15.**
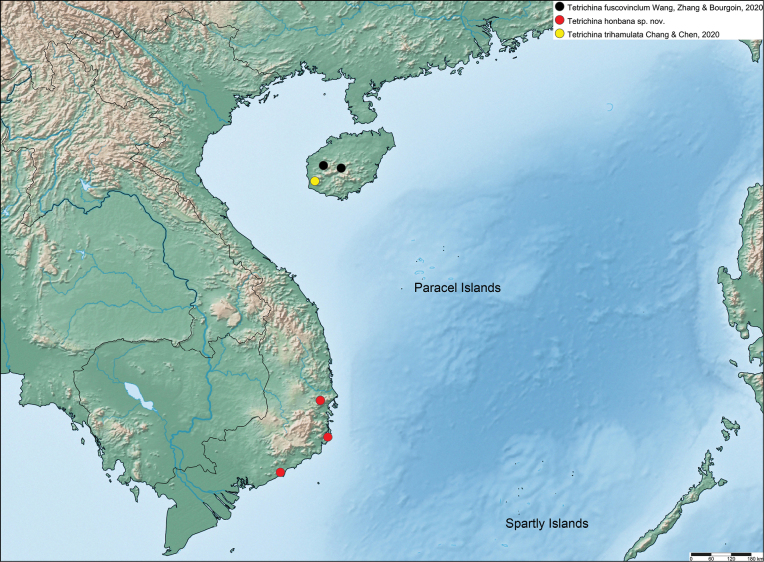
Distribution map of the species of the genus *Tetrichina* Chang & Chen, 2020.

Figs 9, 10, 11, 12, 13, 14, 15

#### Type material.

***Holotype*** ♂, **Vietnam** • Khanh Hoa Province, Hon Ba Nature Reserve; 12°06'46"N, 108°59'52"E; 300–800 m a.s.l.; 8–11 Oct. 2024; J. Constant, J. Bresseel, L. Semeraro and Hoai T.T. Nguyen leg.; VNMN. ***Paratypes*, Vietnam** • 3 ♂♂; Khanh Hoa Province, Nui Chua National Park, 3–6 Oct. 2024; 11°46'28"N 109°11'55"E; 50–700 m; J. Constant, J. Bresseel, L. Semeraro, Hoai T.T. Nguyen leg.; VNMN • 5 ♂♂; same data as for preceding; I.G.: 34893; RBINS • 5 ♂♂, 9 ♀♀; Khanh Hoa Province, Nui Chua National Park; 11°46'28"N 109°11'55"E; 30 Jun.-8 Jul. 2025; 700–1039 m; J. Constant, J. Bresseel, L. Semeraro, Trung Thanh Vu leg.; VNMN • 5 ♂♂, 10 ♀♀; same data as for preceding; I.G.: 35028; RBINS • 2 ♂♂, 1 ♀; Khanh Hoa Province, Nui Chua National Park; 11°46'28"N 109°11'55"E; 30 Jun.-8 Jul. 2025; 50–700 m; J. Constant, J. Bresseel, L. Semeraro, Trung Thanh Vu leg.; VNMN • 2 ♂♂, 2 ♀♀; same data as for preceding; I.G.: 35028; RBINS • 2 ♀♀; Binh Thuan Province, Ta Kou Nature Reserve, temples area; 10°48'50"N 107°53'31"E; 400 m; 18 Jul. 2025; J. Constant, L. Semeraro, Trung Thanh Vu leg.; VNMN • 1 ♂, 1 ♀; same data as for preceding; I.G.: 35028; RBINS.

#### Diagnosis.

*Tetrichina honbana* Constant & Pham, sp. nov. is externally closest to *T. fuscovinclum* Wang, Zhang & Bourgoin, 2020 (see illustrations in Wang et al. 2020: figs 10–18) and *T. trihamulata* Chang & Chen, 2020 (see illustrations in Chang et al. 2020: figs 28–39) but it shows a much shorter lateroventral process of the aedeagus not surpassing midlength of the aedeagus (reaching base of aedeagus in *T. fuscovinclum* and *T. trihamulata*), a more slender and elongate neck of the capitulum than in both other species, and a spine-shaped anterior projection of the laterodorsal process of the dorsal lobe of the periandrium (anterior projection strongly hooked dorsad in both other species [= ‘bidirectional hooked process’ or ‘paired lunate processes’ auctt.]).

#### Description.

***Measurements and ratios***: LT: ♂ (*n* = 9): 4.9 mm (4.7–5.4); ♀ (n = 10): 5.4 (5.2–5.7) LT/BB = 1.6; LTg/BTg = 2.0; LW/BW = 1.3; LV/BV = 0.6; LF/BF = 1.0.

***Head*** (Figs 9A–C, 10A–D). Vertex brown to dark brown in anterior portion and paler, olivaceous in posterior portion, with distinct median carina; ~ 1.6 × as broad as long in midline, distinctly constricted in middle; disc concave; anterior margin weakly projecting anteriad in obtuse angle in middle; posterior margin rather deeply, angularly concave; all margins distinctly raised. Frons variegated olivaceous and brown, moderately convex, smooth (somewhat hairy – short hairs); peridiscal carina distinct in dorsal portion, becoming obsolete towards the ventral; numerous yellowish olivaceous spots between peridiscal carina and lateral and dorsal margins of frons (background darker in upper portion); median carina distinct and complete, extending to base of clypeus; in perpendicular view, dorsal margin concave and lateral margins distinctly sinuate. Genae variegated yellowish olivaceous to brown (slightly paler than frons and vertex) with anteroventral angle weakly projecting anteriad. Clypeus subtriangular, convex, smooth, carinate in middle in basal portion; anteclypeus mostly yellowish olivaceous, sides of anteclypeus and postclypeus blackish brown. Labium olivaceous with last segment longer than broad, shorter than penultimate. Antennae with scape short, ring-shaped, yellowish, and pedicel subcylindrical, yellowish with basal third blackish brown.

***Thorax*** (Figs 9A, 9C, 9D, 10A–D). Pronotum brown with wide yellowish olivaceous transverse band in anterior third; ~ 0.7 × as long as mesonotum in midline; anterior margin carinate, distinctly, angularly protruding anteriorly between eyes; posterior margin weakly curved; no distinct median carina but with impressed point on each side of median line; paradiscal fields very narrow behind eye; paranotal lobes broad, yellowish olivaceous with external margin more or less broadly brown, with distinct black marking under eye, behind antenna and with posteroventral angle rounded. Mesonotum yellowish olivaceous with weak but distinct median carina and obsolete sublateral (peridiscal) carinae; smooth, slightly depressed before scutellum. Tegulae olivaceous brown.

***Tegmina*** (Figs 9A–C, 13). Variegated olivaceous brown, with dark brown marking on clavus, along both sides of Pcu vein, up to ~ 2/3 of clavus; in live specimen, often weakly covered in yellowish waxy (powdery) secretion; veins usually paler than background, main veins elevated, and cross-veins weakly raised and often paler, forming dense reticulum; distinctly convex, and ~ 1.3 × as long as wide in dorsal view when taken together; moderate lateral hump including vein ScP+RA around basal 1/3; moderate but distinct, yellowish epipleuron; clavus closed, reaching 4/5 of tegmen length. Venation: ScP+R rather short; ScP+RA curved in basal portion, reaching costal margin around 4/5 of tegmen length; RP unforked, reaching apical margin; first fork of MP slightly before midlength of tegmen, MP1 with 2–3 terminales, MP2 with 2 terminales; first fork of CuA around midlength of tegmen; Pcu and A1 fused at ~ 2/3 of clavus length, Pcu+A1 reaching apex of clavus; cross-veins more numerous and more strongly marked along costal margin and in distal half of tegmen.

***Hind wings*** (Fig. 10E). Well developed, with three distinct lobes (Sarimini type) more or less equal in width; mostly translucent brown. Venation: ScP+R and CuA furcate; MP simple, straight to nodal line, then upcurved; second branch of CuA fused distally with CuP; Pcu and A1 fused on basal half, Pcu unforked and A2 simple; one transverse vein between second branch of ScP+R and MP, and between MP and first branch of CuA.

***Legs*** (Fig. 9A–E). Yellowish olivaceous, generally paler than tegmina; distal portion of pro- and mesotibiae darkened; all spines of posterior legs black apically. Anterior and median legs slightly flattened dorsoventrally, tibiae more slender than corresponding femora; posteroventral margin of pro- and mesofemora with row of minute teeth; pro- and mesotarsi rather elongate. Metatibiae with two lateral spines in distal portion, and eight apical spines. Metatarsi rather short with first segment shorter than combined length of remaining segments. First metatarsomere with two latero-apical and 9 intermediate, smaller spines arranged in arc. Metatibiotarsal formula: (2) 8 / 11 / 2.

***Abdomen*** (Fig. 9B). Yellowish olivaceous with weak darker band in middle.

***Terminalia*** ♂ (Figs 11, 12). Pygofer (Fig. 11A–D, *Py*) rather massive, ~ 2.5 × as high as long at midheight in lateral view; in lateral view, posterior margin broadly rounded, and dorsal margin rounded; in caudal view suboval, 1.5 × as high as wide; dorsally deeply notched. Gonostyli (Fig. 11A–D, *G*) rather large, moderately convex, with anterodorsal margin more or less vertical up to strongly, abruptly sinuate (back-curved) base of capitulum; ventral margin nearly straight (weakly concave) along most of length; posterior margin distinctly rounded in lateral view and moderately oblique towards base of capitulum; distinctly bisinuate in caudal view (including capitulum); capitulum (Fig. 11A–D, *ca*) with extremely elongate, sinuate neck, strongly projecting anterodorsad in lateral view with shortly pointed apex and with basilateral laminate process projecting lateroventrad, in caudal view curved mesad in distal portion, and with basilateral laminate process directed lateroventrad, forming a short hook. Anal tube (Fig. 11A–D, *An*) elongate, dorsoventrally flattened, and sublanceolate, with lateral margins downcurved beyond anal opening (latter in basal third), rather elongate, ~ 2.0 × as long as wide in dorsal view, more or less evenly narrowing along distal 1/3 towards rounded apex; in lateral view abruptly but rather moderately narrowing at anal opening, then more or less straight with lateral margins expanding in a moderate lobe (widest around 3/4 of anal tube length). Aedeagus (Figs 11E–L, 12, *ae*) symmetrical, moderately curved posterodorsad in lateral view. Ventral lobe of periandrium (Figs 11E–L, 12A–D, *vl*) laminate, laterally produced in rather large upcurved lobe around midlength, then constricted before spatulate distal portion (distinctly narrower than portion with lateral lobes), and with apical margin rounded; slightly shorter than dorsal lobe. Dorsal lobe of periandrium (Figs 11E–F, 11H, 12A–D, *dl*) more or less parallel-sided on most length, slightly narrowing around midlength, with rounded apical margin; in distal portion, laterodorsal process (Figs 11E–L, 12A–D, *ldp*) distinctly produced laterally, showing posterior process with apical hook curved cephalodorsad, and moderately elongate, pointed anterior process (spine-shaped) directed laterad and cephaloventrad; basally to laterodorsal process, distinct lobe produced ventrally and incurved, ventrally covered by ventral lobe of periandrium and forming with latter, a furrow around aedeagus s. str. (Figs 11E–L, 12E–H, *ae*); latter distinctly surpassing dorsal and ventral lobes of periandrium, bifid, each shaft tapering distally to rounded apex; elongate lateroventral processes (Figs 11E–L, 12E–H, *lvp*) arising subapically, distinctly produced laterally, then curved ventrocephalad (following curvature of aedeagus) and with distal 1/2 curved cephalodorsad, and mesad, tapering to pointed apex, reaching to nearly half length of aedeagus (some minor variation in length and curvature was observed). Connective (Fig. 11H, *co*) well developed, corpus connective long, weakly curved in middle portion in lateral view, tectiductus (Fig. 11H, *te*) well developed, conical with wide anterior foramen.

#### Etymology.

The species epithet *honbana* refers to Hon Ba Nature Reserve in Khanh Hoa Province, where the holotype of the new species was collected.

#### Biology.

The specimens were found sitting on leaves (smooth) and small branches of lower vegetation and bushes (Fig. 13) along forest trails, from which they were collected mostly by sweeping, in Hon Ba Nature Reserve along the road, at altitudes from 700 to 800 m a.s.l. (Fig. 14A), in Nui Chua National Park, at ~ 300–700 m a.s.l. (Fig. 14B), and in Ta Kou Nature Reserve, at ~ 400 m, in the forest around the temples at the summit of the mountain.

#### Distribution.

Vietnam, Khanh Hoa Province: Nui Chua National Park and Hon Ba Nature Reserve; Binh Thuan Province, Ta Kou Nature Reserve (Fig. 15).

## Discussion

The extended distribution of the genus *Tetrichina* to Vietnam (the genus was only known from Hainan Island), as well as the addition of a second species to the genus *Dactylissus*, confirms the exceptional richness of the Issidae fauna of Vietnam. The new species of *Dactylissus* is found on the same mountain as the first known species in this genus. Hence, the genus might be endemic to Hon Ba Massif. During our fieldwork in Hon Ba Nature reserve, we were able to find *D. armillarius* Gnezdilov & Soulier-Perkins, 2014 only at the top of the mountain (1300–1400 m), while *D. sinuatus* Constant & Pham, sp. nov. was found exclusively at lower altitudes, with the highest collecting sites ~ 900 m. Although specimen records in MNHN show that both species appeared together at altitudes of 850–872 m in 2013 (Gnezdilov et al. 2014a), we were not able to find any specimen of *D. armillarius* at this altitude in 2024 and 2025.

## Supplementary Material

XML Treatment for Sarimini

XML Treatment for Dactylissus

XML Treatment for Dactylissus sinuatus

XML Treatment for Dactylissus armillarius

XML Treatment for Tetrichina

XML Treatment for Tetrichina honbana

## Additional information

### Use of AI

No use of AI was reported.

### Author ORCIDs

Jérôme Constant https://orcid.org/0000-0003-0254-0863

Thai-Hong Pham https://orcid.org/0000-0002-4763-3679

Tuong Cat Thi Truong https://orcid.org/0000-0002-8593-6283

Hoai Thu Thi Nguyen https://orcid.org/0009-0004-9130-2940

### Data availability

All of the data that support the findings of this study are available in the main text.
